# A first-in-human, phase 1 study of the NEDD8 activating enzyme E1 inhibitor TAS4464 in patients with advanced solid tumors

**DOI:** 10.1007/s10637-020-01055-5

**Published:** 2021-02-09

**Authors:** Noboru Yamamoto, Toshio Shimizu, Kan Yonemori, Shigehisa Kitano, Shunsuke Kondo, Satoru Iwasa, Takafumi Koyama, Kazuki Sudo, Jun Sato, Kenji Tamura, Junichi Tomomatsu, Makiko Ono, Naoki Fukuda, Shunji Takahashi

**Affiliations:** 1 Department of Experimental Therapeutics, National Cancer Center Hospital, 5-1-1 Tsukiji, Chuo-ku, Tokyo, 104-0045 Japan; 2 Present Address: Division of Cancer Immunotherapy Development, Cancer Institute Hospital of the Japanese Foundation for Cancer Research, Tokyo, Japan; 3 Department of Breast and Medical Oncology, National Cancer Center Hospital, Tokyo, Japan; 4 Present Address: Innovative Cancer Center, Shimane University Hospital, Shimane, Japan; 5 Department of Medical Oncology, Cancer Institute Hospital of the Japanese Foundation for Cancer Research, Tokyo, Japan

**Keywords:** Phase 1, Solid tumors, TAS4464, NEDD8 activating enzyme E1 inhibitor, Pharmacokinetics, Safety

## Abstract

*Background* This open-label, phase 1 study investigated TAS4464, a potent NEDD8-activating enzyme inhibitor, in patients with advanced/metastatic solid tumors (JapicCTI-173,488; registered 13/01/2017). The primary objective was dose-limiting toxicities (DLTs). Maximum-tolerated dose (MTD) was investigated using an accelerated titration design. *Methods* The starting 10-mg/m^2^ dose was followed by an initial accelerated stage (weekly dosing; *n* = 11). Based on liver function test (LFT) results, a 14-day, 20-mg/m^2^ dose lead-in period was implemented (weekly dosing with lead-in; *n* = 6). *Results* Abnormal LFT changes and gastrointestinal effects were the most common treatment-related adverse events (AEs). DLTs with 56-mg/m^2^ weekly dosing occurred in 1/5 patients; five patients had grade ≥ 2 abnormal LFT changes at 40- and 56-mg/m^2^ weekly doses. Further dose escalation ceased because of the possibility of severe abnormal LFT changes occurring. DLTs with weekly dosing with lead-in occurred in 1/5 patients at a 56-mg/m^2^ dose; MTD could not be determined because discontinuation criteria for additional enrollment at that particular dose level were met. As no further enrollment at lower doses occurred, dose escalation assessment was discontinued. Serious treatment-related AEs, AEs leading to treatment discontinuation, and DLTs were all related to abnormal LFT changes, suggesting that TAS4464 administration could affect liver function. This effect was dose-dependent but considered reversible. Complete or partial responses to TAS4464 were not observed; one patient achieved prolonged stable disease. *Conclusions* MTD could not be determined due to TAS4464 effects on liver function. Further evaluation of the mechanism of NEDD8-activating enzyme inhibitor-induced abnormal liver function is required. *Trial registration number* JapicCTI-173,488 (registered with Japan Pharmaceutical Information Center). *Registration date* 13 January 2017

**Supplementary Information:**

The online version contains supplementary material available at 10.1007/s10637-020-01055-5.

## Introduction

Cancer is a continued worldwide burden. Based on GLOBOCAN estimates produced by the International Agency for Research on Cancer, an estimated 18 million new cancer cases and 9.6 million cancer deaths occurred in 2018 [1]. As the burden of cancer is expected to increase dramatically, owing to population aging, population growth, and increased prevalence of cancer risks, identifying novel and effective oncolytic treatments is a continued unmet medical need [1, 2].

One approach for the development of novel cancer therapies is treatment targeting the NEDD8 activating enzyme (NAE). Protein homeostasis allows processes from protein synthesis to degradation to be controlled, thereby affecting activities such as cell growth and survival, cellular signaling, and regulation of transcription factors [3, 4]. Ubiquitin-proteasome system-mediated protein degradation is essential for maintaining protein homeostasis in mammalian cells [5, 6]. NEDD8, a ubiquitin-like small protein, has been identified as a regulator of the activity of a group of E3 enzymes within the ubiquitin-proteasome system, including the cullin-RING E3 ubiquitin ligases (CRLs), which control the turnover of several proteins involved in cancer biology [7, 8]. NAE is an essential protein in the NEDD8 conjugation (neddylation) pathway, affecting cancer cell growth and survival through activation of CRLs [8]. This elucidation of the cellular role of NAE in cancer pathogenesis has led to the consideration of NAE as a potential therapeutic target in various malignancies, resulting in the instigation of several early phase clinical studies with the NAE inhibitor MLN4924 (pevonedistat) in both hematologic and solid tumor types [9–12].

TAS4464 is a selective and potent inhibitor of NAE with widespread single-agent antiproliferative effects across diverse tumor cell lines [13]. TAS4464 has shown greater inhibitory effects than MLN4924 in both enzyme assays and cells [13]. In preclinical analyses, the antiproliferative effects of TAS4464 increased in a dose- and time-dependent manner, plateauing after 24 h. Additionally, TAS4464 demonstrated promising antitumor effects in several hematologic and solid tumor xenograft models without notable weight loss [13]. Further, in vitro and in vivo investigations of TAS4464 in multiple myeloma models found that the antitumor activity of TAS4464 occurs through inhibition of nuclear factor κB (NFκB) activation, thereby affecting the canonical and noncanonical NFκB pathways [14].

Based on these preclinical findings, this first-in-human phase 1 study investigated the safety and tolerability, efficacy, pharmacokinetics (PK), and pharmacodynamics of TAS4464 in solid tumors.

## Methods

### Study design and treatment

This non-randomized, open-label, multicenter phase 1 study of TAS4464 (JapicCTI-173,488; registered January 13, 2017) included patients with solid tumors. The highest non-severely toxic dose was derived from a dog toxicology study and was used to calculate a clinical starting dose of 10 mg/m^2^, which corresponds to one-sixth of the human-equivalent dose [15]. The maximum tolerated dose (MTD) of TAS4464 was investigated using an accelerated titration design. Initially, the starting TAS4464 dose was 10 mg/m^2^ and was followed by an initial accelerated stage (i.e., weekly dosing phase) with 100% dose-step increments (level 1: 10 mg/m^2^, level 2: 20 mg/m^2^, level 3: 40 mg/m^2^, and level 4: 56 mg/m^2^). The 100% dose-step increments continued until the occurrence of a grade ≥ 2 drug-related toxicity within Cycle 1; dose-limiting toxicity (DLT) within Cycle 1; the next dose level reaching 60 mg/m^2^; or any safety concerns. Thereafter, 40% increments were employed in the standard stage. Using a 3 + 3 scheme, a maximum of 15 patients were to be enrolled at each dose level.

In the standard stage, three to six patients were to be enrolled, and the dose was escalated in 40% increments if none of the first three patients experienced a DLT. If one patient experienced a DLT, three additional patients were to be enrolled at the same dose level. If two of three or two of six patients experienced a DLT, the dose escalation was to be ceased. Subsequently, and based on the liver function test data during the TAS4464 weekly dose regimen, the study protocol was amended in order to diminish the effect of TAS4464 on liver function; additional discontinuation criteria for patient enrollment were added and a 14-day lead-in period with a TAS4464 dose of 20 mg/m^2^ was implemented throughout all dose levels. Additionally, DLT criteria regarding abnormal liver function and discontinuation criteria for study drug administration were added. The TAS4464 dose level for Day 1 of Cycle 1 was then set to 40 mg/m^2^ (level 3).

Continuous exposure of TAS4464 was not required to achieve cytotoxicity, and the antitumor activity of TAS4464 was statistically significant following a weekly or twice-weekly dosing schedule [13]. Therefore, TAS4464 was administered every 7 days by 1-h intravenous infusion (± 10 min) at the dose assigned to each level. Study treatment was repeated every 28 days. The dosing regimens of each cohort are summarized in Supplementary Fig. 1.

The study was reviewed and approved by the institutional review board at each study site. The study was conducted in compliance with the guidelines provided in the Declaration of Helsinki, Good Clinical Practice, and all applicable local and national regulations. All patients provided written informed consent prior to study participation.

### Patients

The study included patients aged ≥20 years at enrollment who had advanced or metastatic solid tumors and who had a life expectancy of ≥90 days. Patients were to have ≥1 measurable or non-measurable lesion based on Response Evaluation Criteria in Solid Tumors (RECIST) version 1.1 [16], an Eastern Cooperative Oncology Group (ECOG) performance status of 0 or 1, and adequate organ function.

Exclusion criteria were prior use of TAS4464, immunosuppression, serious medical conditions, unresolved toxicities or hypersensitivities to prior treatments, pregnancy, or lactation, or any other reason considered by the investigator to make the patient unsuitable for study participation.

### Outcomes and assessments

The primary objective of the study was to investigate DLTs. Treatment-related adverse events (AEs) during the lead-in period and Cycle 1 were included in the assessment of DLT, which was defined as follows: 1) grade 4 neutropenia lasting >7 days or neutropenia requiring treatment; 2) grade 4 thrombocytopenia or thrombocytopenia requiring blood transfusion; 3) febrile neutropenia; 4) grade ≥ 3 nausea, vomiting, or diarrhea lasting >48 h and uncontrolled by treatment; 5) grade ≥ 3 aspartate aminotransferase (AST) increase or alanine aminotransferase (ALT) increase lasting ≥3 days; or 6) any other grade ≥ 3 non-hematologic toxicity. The MTD was defined as the highest dose level at which <33% of patients experienced a DLT during the lead-in period and Cycle 1.

Secondary objectives included assessment of AEs, treatment-related AEs, objective response rate (ORR), disease control rate (DCR), progression-free survival (PFS), and PK, pharmacodynamic, and pharmacogenomic (PGx) parameters with TAS4464 administration. The National Cancer Institute Common Terminology Criteria for Adverse Events (NCI CTCAE) v4.03 [17] was used to assess the severity of AEs. The Medical Dictionary for Regulatory Activities (MedDRA) version 22.1 was used to classify AEs. RECIST v1.1 [16] was used to determine clinical response, and all computed tomography images were assessed by the physician at the study site after completion of the imaging procedure. The ORR was defined as the percentage of patients with the best overall response of complete response (CR) or partial response (PR). The DCR was the percentage of patients with a best overall response of CR, PR, stable disease (SD), or non-CR/non-progressive disease (PD). PFS was defined as the time from the date of enrollment until the date of investigator-assessed radiological disease progression or death due to any cause, whichever occurred first.

The PK endpoints included maximum plasma concentration (C_max_), area under the concentration–time curve from time zero up to the last observable concentration (AUC_0-last_) and plasma accumulation of TAS4464 after multiple doses. PK parameters were evaluated on Day 1 of Cycle 1 and Day 15 of Cycle 1 in the weekly dosing cohort, as well as on Day 1 of the lead-in period and Day 1 of Cycle 1 in the weekly with lead-in dosing cohort. The evaluation of the relationship between solute carrier organic anion transporter family member 1B1 (*SLCO1B1*) genetic polymorphisms and PK (i.e., the mean and individual clearance [CL] and volume of distribution at steady-state [Vd_ss_] by each *OATP1B1* phenotype) was assessed as the PGx analysis endpoint.

Assessment of pharmacodynamics included evaluation of cullin-NEDD8, p27^Kip1^ protein expression, and cytokines in peripheral blood samples. Expression of cullin-NEDD8 protein in peripheral blood mononuclear cells (PBMC) was analyzed using an enzyme-linked immunosorbent assay (ELISA). For the weekly dosing cohorts, changes from baseline in cullin-NEDD8 complexes in the PBMC on Day 1 and Day 2 of Cycle 1 as measured by ELISA were evaluated. For the weekly dosing with lead-in period cohorts, changes from baseline in cullin-NEDD8 complexes on Day 2 were evaluated. Expression of p27^Kip1^ protein (cyclin-dependent kinase inhibitor 1B), a substrate of CRL, was determined by immunohistochemistry (IHC). p27^Kip1^ IHC was performed on biopsy tumor specimens collected at baseline and Day 2 of Cycle 1, and their staining intensities were assessed using H-Scoring, with the scores compared between baseline and Day 2 of Cycle 1. To investigate the correlations between cytokines and liver function laboratory test abnormalities, concentrations of 10 cytokines in peripheral blood, including tumor necrosis factor-alpha (TNF-α), interferon gamma (IFNγ), interleukin (IL)-12p40, IL-12p70, IL-17A, IL-1α, IL-1β, IL-2, IL-6, and IL-8, were measured by the Luminex® 100/200TM System (Luminex Corp; Austin, TX, USA) using the plasma of peripheral blood samples collected at baseline, Day 8 of the lead-in period, Day 1, Day 3, and Day 15 of Cycle 1.

### Statistical analysis

The analysis populations included in the study are summarized in the Supplementary Appendix. DLTs were recorded by incidence and 95% confidence intervals (CI). Median PFS and 95% CIs were calculated using Kaplan–Meier methodology. Best overall response was tabulated, and the ORR and DCR with 95% CIs were calculated by dose level. PK parameters were evaluated using non-compartmental methods. For the pharmacodynamic parameter of cullin-NEDD8 in PBMC, summary statistics and a scatter plot of the percentage change from baseline were produced. Missing data were not imputed. The analyses did not include tests for significance; thus, it was not necessary to adjust for multiplicity. SAS software v9.4 (SAS Institute Inc., Cary NC, USA) was used for all statistical processing.

## Results

### Patients

In this study, 17 patients were enrolled from February 2017 to August 2019 in two Japanese institutions. One patient was assigned to weekly dosing level 1 (10 mg/m^2^), two patients to weekly dosing level 2 (20 mg/m^2^), three patients to weekly dosing level 3 (40 mg/m^2^), five patients to weekly dosing level 4 (56 mg/m^2^), and three patients each to weekly dosing with lead-in level 3 (20 → 40 mg/m^2^) and level 4 (20 → 56 mg/m^2^) (Table 1). One patient who did not receive the study drug was excluded from the full analysis set. The demographic and disease characteristics of the study population are summarized in Table 2. The median age was 58.0 years.

**Table 1:** Patient flow

Population	Weekly dosing					Weekly dosing with lead-in period			
	Level 1 (10 mg/m^2^) *n* (%)	Level 2 (20 mg/m^2^) *n* (%)	Level 3 (40 mg/m^2^) *n* (%)	Level 4 (56 mg/m^2^) *n* (%)	Total *N* (%)	Level 3 (20 → 40 mg/m^2^) *n* (%)	Level 4 (20 → 56 mg/m^2^) *n* (%)	Total *n* (%)	Total *N* (%)
All enrolled patients	1	2	3	5	11	3	3	6	17
FAS	1 (100.0)	1 (50.0)	3 (100.0)	5 (100.0)	10 (90.9)	3 (100.0)	3 (100.0)	6 (100.0)	16 (94.1)
Patients excluded from FAS	0	1 (50.0)	0	0	1 (9.1)	0	0	0	1 (5.9)
All treated patients	1 (100.0)	1 (50.0)	3 (100.0)	5 (100.0)	10 (90.9)	3 (100.0)	3 (100.0)	6 (100.0)	16 (94.1)
Untreated patients	0	1 (50.0)	0	0	1 (9.1)	0	0	0	1 (5.9)
DLT-evaluable patients	1 (100.0)	1 (50.0)	3 (100.0)	5 (100.0)	10 (90.9)	3 (100.0)	2 (66.7)	5 (83.3)	15 (88.2)
DLT-unevaluable patients	0	1 (50.0)	0	0	1 (9.1)	0	1 (33.3)	1 (16.7)	2 (11.8)
PK-evaluable patients	1 (100.0)	1 (50.0)	3 (100.0)	5 (100.0)	10 (90.9)	3 (100.0)	3 (100.0)	6 (100.0)	16 (94.1)
PK-unevaluable patients	0	1 (50.0)	0	0	1 (9.1)	0	0	0	1 (5.9)
Pharmacodynamic-evaluable patients	1 (100.0)	1 (50.0)	3 (100.0)	5 (100.0)	10 (90.9)	3 (100.0)	2 (66.7)	5 (83.3)	15 (88.2)
Pharmacodynamic-unevaluable patients	0	1 (50.0)	0	0	1 (9.1)	0	1 (33.3)	1 (16.7)	2 (11.8)
PGx-evaluable patients	1 (100.0)	1 (50.0)	3 (100.0)	5 (100.0)	10 (90.9)	3 (100.0)	3 (100.0)	6 (100.0)	16 (94.1)
PGx-unevaluable patients	0	1 (50.0)	0	0	1 (9.1)	0	0	0	1 (5.9)

*DLT* dose-limiting toxicity, *FAS* full analysis set, *PGx* pharmacogenomic, *PK* pharmacokinetics

**Table 2:** Patient demographics and disease characteristics

	Weekly dosing				Weekly dosing with lead-in		Total (*N* = 16)
	Level 1 (*n* = 1)	Level 2 (*n* = 1)	Level 3 (*n* = 3)	Level 4 (*n* = 5)	Level 3 (*n* = 3)	Level 4 (*n* = 3)	
Sex							
Male	0	0	2 (66.7)	2 (40.0)	1 (33.3)	2 (66.7)	7 (43.8)
Female	1 (100.0)	1 (100.0)	1 (33.3)	3 (60.0)	2 (66.7)	1 (33.3)	9 (56.3)
Age (years)							
Median (min, max)	57.0 (57, 57)	65.0 (65, 65)	64.0 (45, 69)	51.0 (23, 64)	45.0 (36, 67)	72.0 (30, 73)	58.0 (23, 73)
ECOG performance status							
0	1 (100.0)	0	3 (100.0)	4 (80.0)	2 (66.7)	3 (100.0)	13 (81.3)
1	0	1 (100.0)	0	1 (20.0)	1 (33.3)	0	3 (18.8)
Tumor type							
Pancreatic	0	0	0	0	0	1 (33.3)	1 (6.3)
Rectal	0	0	1 (33.3)	1 (20.0)	1 (33.3)	0	3 (18.8)
Soft tissue sarcoma	1 (100.0)	0	1 (33.3)	0	0	0	2 (12.5)
Uterine	0	0	0	0	0	1 (33.3)	1 (6.3)
Other	0	1 (100.0)	1 (33.3)	4 (80.0)	2 (66.7)	1 (33.3)	9 (56.3)

Data are *n* (%) unless otherwise stated

*ECOG* Eastern Cooperative Oncology Group

### Safety

The treatment-related AEs occurring in at least two patients are summarized in Table 3. The most common (occurring in ≥30% of patients) all-grade treatment-related AEs were ALT increased (68.8%), AST increased (62.5%), nausea (43.8%), alkaline phosphatase (ALP) increased (43.8%), and decreased appetite (31.3%). Grade ≥ 3 treatment-related AEs occurring in ≥30% of patients were ALT increased and AST increased (31.3% each).

**Table 3:** Treatment-related adverse events

	Weekly dosing								Weekly dosing with lead-in				Total (*N* = 16)	
Treatment-related adverse event, *n* (%)	Level 1 (*n* = 1)		Level 2 (*n* = 1)		Level 3 (*n* = 3)		Level 4 (*n* = 5)		Level 3 (*n* = 3)		Level 4 (*n* = 3)			
	All-grade	Grade ≥ 3	All-grade	Grade ≥ 3	All-grade	Grade ≥ 3	All-grade	Grade ≥ 3	All-grade	Grade ≥ 3	All-grade	Grade ≥ 3	All-grade	Grade ≥ 3
ALT increased	1 (100.0)	0	1 (100.0)	0	1 (33.3)	1 (33.3)	5 (100.0)	3 (60.0)	1 (33.3)	0	2 (66.7)	1 (33.3)	11 (68.8)	5 (31.3)
AST increased	1 (100.0)	0	0	0	1 (33.3)	1 (33.3)	4 (80.0)	3 (60.0)	2 (66.7)	0	2 (66.7)	1 (33.3)	10 (62.5)	5 (31.3)
Nausea	0	0	0	0	2 (66.7)	0	4 (80.0)	0	1 (33.3)	0	0	0	7 (43.8)	0
ALP increased	0	0	0	0	2 (66.7)	0	4 (80.0)	1 (20.0)	0	0	1 (33.3)	0	7 (43.8)	1 (6.3)
Decreased appetite	0	0	0	0	2 (66.7)	0	2 (40.0)	0	1 (33.3)	0	0	0	5 (31.3)	0
Vomiting	0	0	0	0	1 (33.3)	0	2 (40.0)	0	1 (33.3)	0	0	0	4 (25.0)	0
GGT increased	0	0	0	0	1 (33.3)	0	3 (60.0)	3 (60.0)	0	0	0	0	4 (25.0)	3 (18.8)
Anemia	0	0	0	0	0	0	1 (20.0)	0	1 (33.3)	0	1 (33.3)	0	3 (18.8)	0
Diarrhea	0	0	0	0	1 (33.3)	0	2 (40.0)	0	0	0	0	0	3 (18.8)	0
Malaise	0	0	0	0	1 (33.3)	0	2 (40.0)	0	0	0	0	0	3 (18.8)	0
Blood bilirubin increased	0	0	0	0	1 (33.3)	0	2 (40.0)	0	0	0	0	0	3 (18.8)	0
WBC decreased	0	0	0	0	1 (33.3)	0	1 (20.0)	1 (20.0)	0	0	1 (33.3)	0	3 (18.8)	1 (6.3)
Rash	0	0	0	0	0	0	2 (40.0)	0	0	0	1 (33.3)	0	3 (18.8)	0
Fatigue	0	0	0	0	1 (33.3)	0	1 (20.0)	0	0	0	0	0	2 (12.5)	0
Pyrexia	0	0	0	0	0	0	0	0	2 (66.7)	0	0	0	2 (12.5)	0
Hypoalbuminemia	1 (100.0)	0	0	0	1 (33.3)	0	0	0	0	0	0	0	2 (12.5)	0
Neuropathy peripheral	0	0	0	0	0	0	2 (40.0)	0	0	0	0	0	2 (12.5)	0

This table shows adverse drug reactions occurring in at least two patients

*ALP* alkaline phosphatase, *ALT* alanine aminotransferase, *AST* aspartate aminotransferase, *GGT* gamma-glutamyl transferase, *WBC* white blood cell

Four serious AEs were reported in three patients. Of these, ALT increased occurring in one patient (weekly dosing level 4) was assessed by the investigator to be treatment related, which resolved following TAS4464 dose reduction. AEs leading to treatment discontinuation or dose reduction were observed in one patient each: AST and ALT increased (weekly dosing with lead-in period level 4) and ALT increased (weekly dosing level 4), respectively. No deaths or fatal AEs occurred within the study period. No patients presented with drug-induced liver injury, as determined by Hy’s law criteria (ALT or AST elevation >3 × upper limit of normal [ULN], total bilirubin elevation >2 × ULN, and ALP elevation <2 × ULN) with doses of up to 56 mg/m^2^.

Among the DLT-evaluable population, a DLT event (i.e., grade 4 ALT increased) in the TAS4464 weekly dosing regimen occurred in one of five patients at dose level 4 (56 mg/m^2^). Grade ≥ 2 abnormal liver function tests did not occur at the 10 or 20 mg/m^2^ weekly doses, but did occur in five patients at the 40 and 56 mg/m^2^ weekly doses. Patient enrollment at dose level 4 was discontinued because of the possibility of severe abnormal liver function tests, and further dose escalation was stopped. Therefore, the MTD for the TAS4464 weekly dosing regimen could not be determined.

During the weekly dosing with lead-in period, DLT events (i.e., grade 3 ALT increased and grade 3 AST increased) occurred in one of five patients at dose level 4 (56 mg/m^2^). The MTD could not be determined because the discontinuation criteria for additional patient enrollment (i.e., > 8 × ULN for AST or ALT) were met at TAS4464 level 4 of the weekly lead-in dosing regimen, and there was no further enrollment at lower dose levels. Therefore, the dose-escalation assessment of TAS4464 was discontinued.

### Clinical response

The clinical response findings from the weekly dosing and weekly dosing with lead-in period regimens are summarized in Table 4. Within the weekly dosing cohort, no patients showed a CR or PR as the best overall response, while five of 10 patients achieved SD, resulting in a DCR of 50.0% (95% CI 18.7–81.3). The primary tumors in patients with SD were soft tissue sarcoma, rectal, cervical, thymic, and tumor of unknown primary (one patient each). The TAS4464 dose levels in these patients were level 1 (10 mg/m^2^; one patient), level 2 (20 mg/m^2^; one patient), level 3 (40 mg/m^2^; one patient), and level 4 (56 mg/m^2^; two patients). One patient in weekly dosing level 2, with a tumor of unknown primary, achieved an SD duration of at least 6 months. Overall, the median PFS was 3.7 months (95% CI 1.0–7.0).

**Table 4:** Clinical response

	Weekly dosing					Weekly dosing with lead-in			Total (*N* = 16) *n* (%)
	Level 1 (10 mg/m^2^) (*n* = 1) *n* (%)	Level 2 (20 mg/m^2^) (*n* = 1) *n* (%)	Level 3 (40 mg/m^2^) (*n* = 3) *n* (%)	Level 4 (56 mg/m^2^) (n = 5) *n* (%)	Total (*N* = 10) *n* (%)	Level 3 (20 → 40 mg/m^2^) (*n* = 3) *n* (%)	Level 4 (20 → 56 mg/m^2^) (*n* = 3) *n* (%)	Total (*n* = 6) *n* (%)	
SD	1 (100.0)	1 (100.0)	1 (33.3)	2 (40.0)	5 (50.0)	0 (0.0)	0	0	5 (31.3)
PD	0	0	2 (66.7)	3 (60.0)	5 (50.0)	3 (100.0)	2 (66.7)	5 (83.3)	10 (62.5)
Not evaluable	0	0	0	0	0	0	1 (33.3)	1 (16.7)	1 (6.3)
Response rate^a^	0	0	0	0	0	0	0	0	0
95% CI	–	–	–	–	–	–	–	–	–
Disease control rate^b^	1 (100.0)	1 (100.0)	1 (33.3)	2 (40.0)	5 (50.0)	0	0	0	5 (31.3)
95% CI	[2.5, 100.0]	[2.5, 100.0]	[0.8, 90.6]	[5.3, 85.3]	[18.7, 81.3]	–	–	–	[11.0, 58.7]

^a^(CR + PR)

^b^(CR + PR + SD + non-CR/non-PD)

*CI* confidence interval, *CR* complete response, *PD* progressive disease*, PR* partial response, *SD* stable disease

In the weekly dosing with lead-in period cohort, no patients showed CR, PR, or SD as the best overall response, and the median PFS was 1.5 months (95% CI 0.5–2.9).

### Pharmacokinetics

**Fig. 1:**
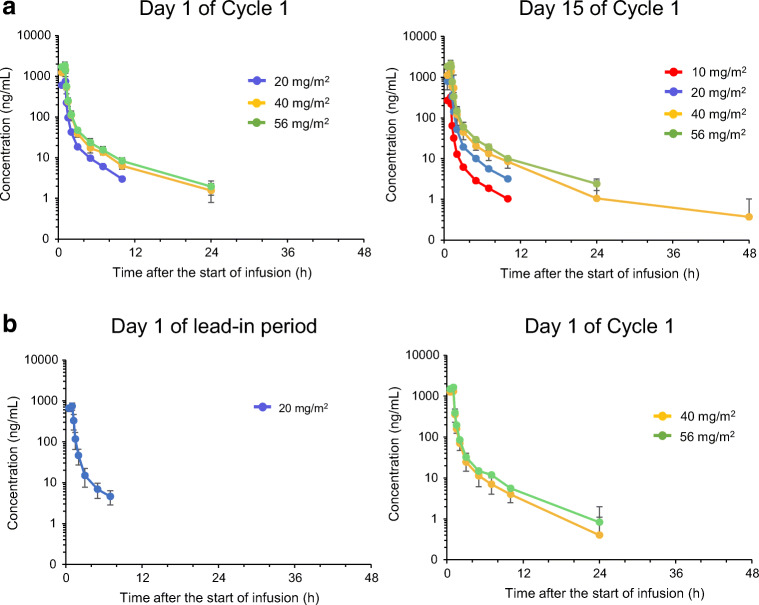
Mean plasma concentration-time profiles of TAS4464 with **a** a weekly dosing regimen and **b** a weekly dosing with lead-in period. Each point represents the mean + standard deviation. The scheduled times were used for plotting

The mean plasma concentration–time profiles of TAS4464 in each dosing regimen are illustrated in Fig. 1. The accumulation ratios of C_max_ [R (C_max_)] and AUC_0-last_ [R (AUC_0-last_)] were close to unity (1.0), suggesting no plasma accumulation of TAS4464 (data not shown).

### Pharmacogenomics

The relationship between *SLCO1B1* genetic polymorphisms and TAS4464 PK was investigated. The mean and individual CL and Vd_ss_ by each *OATP1B1* phenotype assigned by the measured *SLCO1B1* genotypes were investigated, and no obvious differences were observed (data not shown).

### Pharmacodynamics

The pharmacodynamics of cullin-NEDD8 in PBMC were assessed. Ten patients were evaluated in the weekly dosing cohort, including one patient in level 1 (10 mg/m^2^), one in level 2 (20 mg/m^2^), three in level 3 (40 mg/m^2^), and five in level 4 (56 mg/m^2^). Five patients were evaluated in the weekly dosing with lead-in period cohort, including three in level 3 (20 → 40 mg/m^2^) and two in level 4 (20 → 56 mg/m^2^). As a result, cullin-NEDD8 complex measurements at Day 1 of Cycle 1 were lower than those at baseline in all patients. However, no dose-dependency was observed. The measurements on Day 2 of Cycle 1 were lower than those at baseline, with the exception of two patients, but dose dependency was not observed.

Tumor specimens from three patients were evaluated for p27^Kip1^ immunostaining. The expression of p27^Kip1^ in specimens collected on Day 2 of Cycle 1 was almost unchanged or decreased compared with those at baseline (data not shown). No p27^Kip1^ accumulation effect in tumors was observed as a result of NAE inhibition by TAS4464 administration.

Cytokine expression in peripheral blood was also assessed. Ten cytokines were evaluated in five patients, including three patients in level 3 (20 → 40 mg/m^2^) and two in level 4 (20 → 56 mg/m^2^) of the weekly dosing with lead-in period cohort. For one patient in the weekly dosing with lead-in period cohort at level 3, the following seven cytokines reached their peak over the assessment period on Day 3 of Cycle 1 with expression levels more than four times higher than at baseline: IL-1β, IFNγ, IL-12p40, IL-12p70, IL-17A, IL-1α, and IL-2. Similar changes were not observed in the other patients assessed. Thus, no changes in the expression levels of the 10 cytokine groups, including TNF-α, were observed under the administration of TAS4464.

## Discussion

In this first-in-human phase 1 study, the MTD of TAS4464 could not be determined because of the possibility of severe abnormal changes in liver function tests occurring with further dose escalation.

Hepatotoxicity with NAE inhibitors has been reported previously. Clinical trials of another NAE inhibitor under development (MLN4924) have been conducted, and abnormal liver function tests (i.e., ALT and AST increased) have been described as one of the major AEs [9–12]. Although the mechanism of development of hepatoxicity with NAE inhibitors has not been elucidated, a study in rats found no increases in AST, ALT, or sorbitol dehydrogenase following MLN4924 administration. Increases in laboratory values for these items have been reported for the combination of TNF-α and MLN4924 [18, 19]. In a study of its anti-inflammatory effects, MLN4924 has been observed to suppress the production of TNF-α in cells derived from macrophages, a major producer of TNF-α [19]. MLN4924 inhibits the production of cytokines, including TNF-α, and its efficacy in bleomycin-induced idiopathic pulmonary fibrosis models has been shown [20].

In canine toxicology studies, toxic liver findings were not observed using TAS4464 without a *N,N*-dimethylacetamide group, but were observed using TAS4464 with *N,N*-dimethylacetamide. Abnormal liver function tests were also observed in TAS4464 non-clinical studies combining TAS4464 with TNF-α (unpublished data).

Based on the weekly dosing results of TAS4464 in our phase 1 study, no abnormalities in liver function tests were seen at TAS4464 doses of ≤20 mg. Additionally, an abnormal increase in liver function test values was not observed after the third dose. As a result of these findings, it was expected that administrating a low dose of TAS4464 without abnormal liver function test values would suppress NFκB-mediated TNF-α production, which would alleviate the effects on hepatocytes when subsequent high doses of TAS4464 are administrated. The study protocol was amended and a 14-day lead-in period (i.e., a TAS4464 dose of 20 mg/m^2^ given on Day 1 and Day 8 in which no grade ≥ 2 abnormal liver function test values were observed) was newly added to the schedule. In the weekly with lead-in period at level 4 (20 → 56 mg/m^2^), abnormal liver function test values (i.e., grade 3 elevated ALT and AST values) were observed, meeting the discontinuation criteria for additional patient enrollment. Subsequent patient enrollment at that particular dose level was discontinued, and the safety of TAS4464 administration at the weekly with lead-in dosing of level 4 (56 mg/m^2^) or higher has not been confirmed.

Serious AEs considered to be related to TAS4464, AEs requiring discontinuation of study treatment, and DLTs were all related to abnormal changes in liver function test values, suggesting that TAS4464 administration may affect liver function. All events recovered quickly or resolved, and the effects on liver function were, therefore, considered to be reversible. These data suggest that humans may be highly susceptible to liver function test abnormalities, and this toxicity may be a class effect of NAE inhibitors considering the similar reports with MLN4924. However, no abnormal findings in liver function test values or histopathological examination were observed in non-clinical studies, limiting the ability to predict toxicity in phase 1 oncology clinical trials [21].

The PK of TAS4464 was also assessed in this study. The C_max_ and AUC of TAS4464 generally increased with increasing dose within the dose range of 10–56 mg/m^2^. No obvious accumulation of TAS4464 was found after multiple administrations. The exploratory assessment suggested that the PK of TAS4464 is not affected by *SLCO1B1* genetic polymorphisms. Grade ≥ 2 abnormal liver function tests did not occur at ≤20 mg/m^2^ weekly doses but occurred at weekly doses of ≥40 mg/m^2^; no clear correlations were found between PK parameters and abnormal liver function test values likely because of the small sample size. The reduction in cullin-NEDD8 complex abundance in the PBMC measured as a pharmacodynamic marker suggested an NAE inhibitory effect by TAS4464 administration, but there was no correlation with clinical response or dose-dependency. Additionally, no accumulation of p27^Kip1^ in tumors as a result of NAE inhibition by TAS4464 administration was observed. No correlations were observed between the serum levels of cytokines, including TNF-α, and laboratory abnormalities of liver functions.

The efficacy of TAS4464 could not be evaluated in this study. This is because 10–56 mg/m^2^ of TAS4464 (AUC ranging from 334 to 3033 ng•hr./mL) could not reach the levels needed to be pharmacologically effective (calculated based on nonclinical studies) owing to liver function disorders.

In conclusion, although a dose-dependent effect on liver function tests was observed after TAS4464 administration, this effect was considered to be reversible. Other than abnormal liver function tests, no severe AEs that could affect the administration of TAS4464 (i.e., drug administration suspension, discontinuation, or delay) were observed. Although pharmacodynamic data suggested possible NAE inhibition by TAS4464, our data did not show sufficient clinical benefit with the doses administered (up to 56 mg/m^2^). MTD could not be determined due to the effect of TAS4464 on liver function.

## Supplementary Information

ESM 1 (PDF 253 kb)

## Data Availability

Data will not be shared according to the sponsor policy on data sharing.
