# Supplementary material for: A first-in-human, phase 1 study of the NEDD8 activating enzyme E1 inhibitor TAS4464 in patients with advanced solid tumors
Source: Invest New Drugs. 2021 Feb 9;39(4):1036–46. doi: 10.1007/s10637-020-01055-5 (PMC8279981; doi:10.1007/s10637-020-01055-5)
Supplement: Supplementary file 1 — (PDF 253 kb) [file 10637_2020_1055_MOESM1_ESM.pdf]

Supplementary Appendix

Supplementary Fig. 1 Dosing regimens

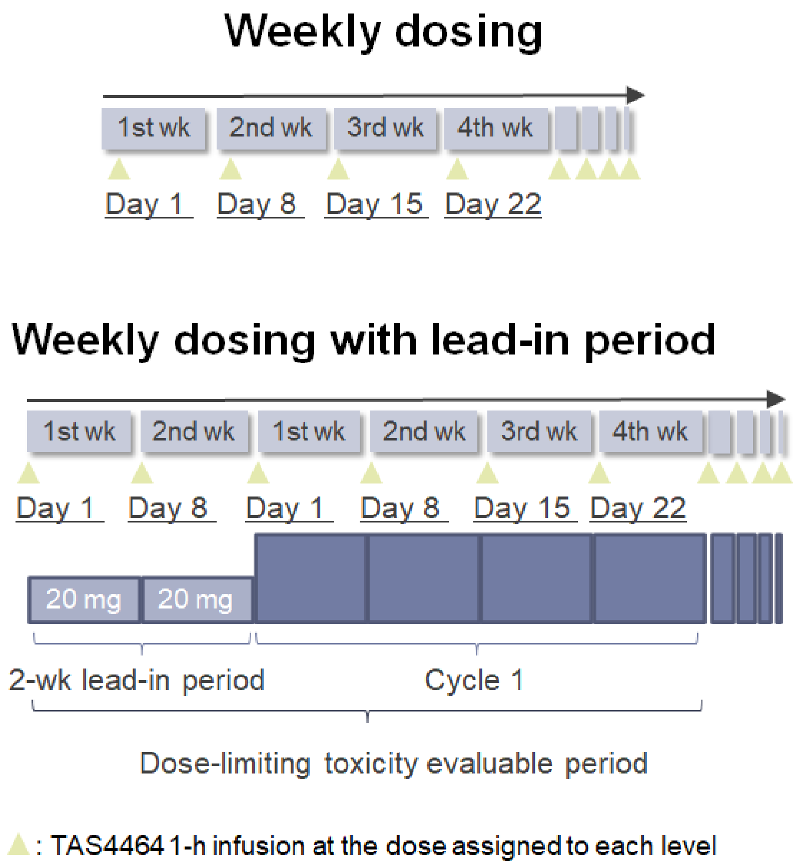

wk, week

Supplementary Methods

Analysis populations

The DLT-evaluable population included all patients in the all-treated population in the weekly dosing and weekly dosing with lead-in period cohorts apart from patients who received prohibited medication or therapies in the lead-in period and Cycle 1, who experienced a DLT, whose administration was less than three out of four times in Cycle 1 or who did not administer both doses in the lead-in period or who did not experience a DLT, and patients who had not undergone any specified tests or observations in the lead-in period and Cycle 1.

The full-analysis set, which was used for assessment of efficacy, included all treated patients who were evaluated for at least one efficacy endpoint after administration of TAS4464.

The PK-evaluable population included all patients in the all-treated population who had evaluable concentrations of TAS4464 in plasma and urine.

The pharmacodynamic-evaluable population included all patients in the all-treated population who had evaluable data for cullin-NEDD8 in the peripheral blood mononuclear cells (PBMC) and $p27^{Kip1}$ in tumor biopsy samples.

The PGx evaluable population included all patients in the PK-evaluable population who were determined to be evaluable for *SLCO1B1* polymorphisms.
